# The effect of smoking on survival in lung carcinoma patients with brain metastasis: a systematic review and meta-analysis

**DOI:** 10.1007/s10143-022-01832-1

**Published:** 2022-07-14

**Authors:** Shreya Chawla, Ishaan A. Tewarie, Qingwei O. Zhang, Alexander F. C. Hulsbergen, Rania A. Mekary, Marike L. D. Broekman

**Affiliations:** 1 Faculty of Life Sciences and Medicine, King’s College London, London, WC2R 2LS UK; 2 Computational Neuroscience Outcomes Center (CNOC), Department of Neurosurgery, Brigham and Women’s Hospital, Harvard Medical School, 75 Francis Street, Boston, MA 02115 USA; 3 Department of Neurosurgery, Leiden University Medical Center, Albinusdreef 2, 2333ZA Leiden, Zuid-Holland The Netherlands; 4 Department of Neurosurgery, Haaglanden Medical Center, Lijnbaan 32, 2512VA The Hague, Zuid-Holland The Netherlands; 5 Faculty of Medicine, Imperial College London, London, SW7 2AZ UK; 6 Department of Pharmaceutical Business and Administrative Sciences, School of Pharmacy, Massachusetts College of Pharmacy and Health Sciences (MCPHS) University, 179 Longwood Avenue, Boston, MA 02115 USA; 7 Department of Neurology, Massachusetts General Hospital, Harvard Medical School, 55 Fruit Street, Boston, MA 02114 USA

**Keywords:** Tobacco, Smoking status, Brain metastases, Survival, Meta-analysis

## Abstract

The effects of smoking on survival in BM patients have yet to be reviewed and meta-analysed. However, previous studies have shown that smokers had a greater risk of dying from lung cancer compared to non-smokers. This meta-analysis, therefore, aimed to analyse the effects of cigarette smoking on overall survival (OS) and progression-free survival (PFS) in lung cancer BM patients. PubMed, Embase, Web of Science, Cochrane and Google Scholar were searched for comparative studies regarding the effects of smoking on incidence and survival in brain metastases patients up to December 2020. Three independent reviewers extracted overall survival (OS) and progression-free survival data (PFS). Random-effects models were used to pool multivariate-adjusted hazard ratios (HR). Out of 1890 studies, fifteen studies with a total of 2915 patients met our inclusion criteria. Amongst lung carcinoma BM patients, those who were smokers (ever or yes) had a worse overall survival (HR: 1.34, 95% *CI* 1.13, 1.60, *I*2: 72.1%, p-heterogeneity < 0.001) than those who were non-smokers (never or no). A subgroup analysis showed the association to remain significant in the ever/never subgroup (HR: 1.34, 95% *CI* 1.11, 1.63) but not in the yes/no smoking subgroup (HR: 1.30, 95% *CI* 0.44, 3.88). This difference between the two subgroups was not statistically significant (*p* = 0.91). Amongst lung carcinoma BM patients, smoking was associated with a worse OS and PFS. Future studies examining BMs should report survival data stratified by uniform smoking status definitions.

**Supplementary Information:**

The online version contains supplementary material available at 10.1007/s10143-022-01832-1.

## Introduction

Brain metastases (BMs) have the highest incidence of all central nervous system tumours in adult patients [1]. BMs disseminate most frequently from lung carcinomas (40–50%), breast carcinomas (15–25%) or melanoma (5–20%) [4, 15, 40]. BM survival has remained abysmal despite therapeutic and diagnostic advancements, leading to severe deterioration in function and quality of life [35].

Tobacco use, specifically cigarette smoking, is a major cause of preventable death and morbidity [39]. Smoking has been demonstrated as a risk factor for numerous cancers, including lung, liver and otolaryngological cancers. Both malignancy incidence and therapeutic response to anti-oncological agents are demonstrated to be altered due to smoking [42]. Previous studies examining the effect of smoking on cancer patients have often restricted the included patient population to non-metastatic patients [36, 37]. Smoking cessation may be less likely emphasised for many metastatic patients, especially those who are considered incurable and those with have limited expected survival time.

The effects of smoking on survival in BM patients have yet to be reviewed and meta-analysed. This meta-analysis, therefore, aimed to analyse the effects of cigarette smoking on overall survival (OS) and progression-free survival (PFS) in lung cancer BM patients.

## Materials and methods

The systematic review was conducted in accordance with the 2020 Preferred Reporting Items for Systematic Reviews and Meta-Analyses (PRISMA) checklist, and all steps of the PRISMA checklist were completed [34].

### Inclusion and exclusion criteria

Our inclusion criteria aimed to identify comparative observational studies which analysed the effect of smoking on overall survival (OS) and/or progression-free survival (PFS) in lung cancer patients with brain metastases. If papers included more than one hazard ratio that met inclusion criteria, all would be included as long as the patient populations did not overlap. Search results were limited to English-language studies with > 10 participants.

### Search strategy

The electronic databases MEDLINE (Pubmed), Web of Science, EMBASE, Cochrane and Google Scholar were searched using search terms related to smoking, intracranial metastases originating from lung carcinoma and patient survival (Supplementary materials, Appendix 1); studies published up until 31st December 2020 were included in our screening. Three independent authors (SC, IT, QZ) initially screened the title and abstracts against the inclusion and exclusion criteria. Subsequently, full text articles were retrieved and reviewed against eligibility criteria (SC, IT, QZ), with disagreements resolved through discussion with each other or a fourth author (AH).

### Data extraction

Relevant data from each included study were extracted by three independent authors (SC, IT, QZ) as follows: (1) study characteristics, (2) cohort demographics, (3) smoking status, (4) primary tumour characteristics, (5) BM characteristics, (6) treatment characteristics and (7) outcomes (OS, PFS).

### Quality assessment

Two independent authors (SC, QZ) assessed the quality of each included article using the Newcastle Ottawa Scale [46] for observational studies. Any disagreements were discussed and resolved amongst the authors; if consensus was not reached, a third author (IT) gave a final judgement. The Newcastle Ottawa Scale assesses the domains of subject selection (4 points), comparability (2 points) and assessment of outcome (3 points) for a total of 9 points. The score was interpreted as 0–3 points = “poor quality”, 4–6 points = “fair quality” and 7–9 points = “good quality”. Studies were penalised in the “selection of the non-exposed cohort” category if they lumped never and former smokers in one category when reporting exposure to smoking.

### Data analysis

#### Statistical methods and analysis

We only included studies providing a multivariate hazard ratio (HR) in our main analysis. Pooled point estimates and their 95% confidence interval were calculated in the meta-analysis using the DerSimonian and Laird random-effects model [11] to account for inter-study variation. Subgroup analysis was performed to tease out heterogeneity in how smoking was reported across studies. Across the fifteen studies, smoking status was reported as either ever vs. never (12 studies) or yes vs. no (3 studies). If a study provided both intracranial and extracranial PFS as opposed to giving an overall PFS, only the results for intracranial PFS were included in the analysis to avoid patient overlap. Unless otherwise specified, a two-sided *p* value of < 0.05 was considered statistically significant. Data analysis was performed in RStudio v. 1.2.1335 (R Core Team, Vienna, Australia) using the package meta [3].

### Sensitivity analysis

We performed sensitivity analyses which included (1) pooling the multivariate studies with the outlier removed and (2) pooling studies that provided a univariate HR. Similar to the multivariate analysis, a subgroup analysis was performed to compare studies reporting smoking as ever/never and yes/no.

#### Heterogeneity assessment and analysis

The degree of heterogeneity amongst studies was determined using the *p* value for the Cochrane *Q* test (statistically significant *p* value < 0.1) [20] and Higgins’ and Thompson’s *I*^2^ value [19]. Degree of heterogeneity was reported to be low, medium and high with *I*^2^ values of 25%, 50% and 75%, respectively [10].

#### Small study effect

Potential small study effects were identified using a funnel plot for visual determination of asymmetry, as well as Egger’s test for statistical significance [14]. When small study effects were indicated, the trim-and-fill method was used to impute the potentially missing studies and recalculate the imputed pooled effect estimate, whilst acknowledging the limitation of such a method, which assumes the source of asymmetry to be due solely to small study effect and not to other reasons.

## Results

**Fig. 1:**
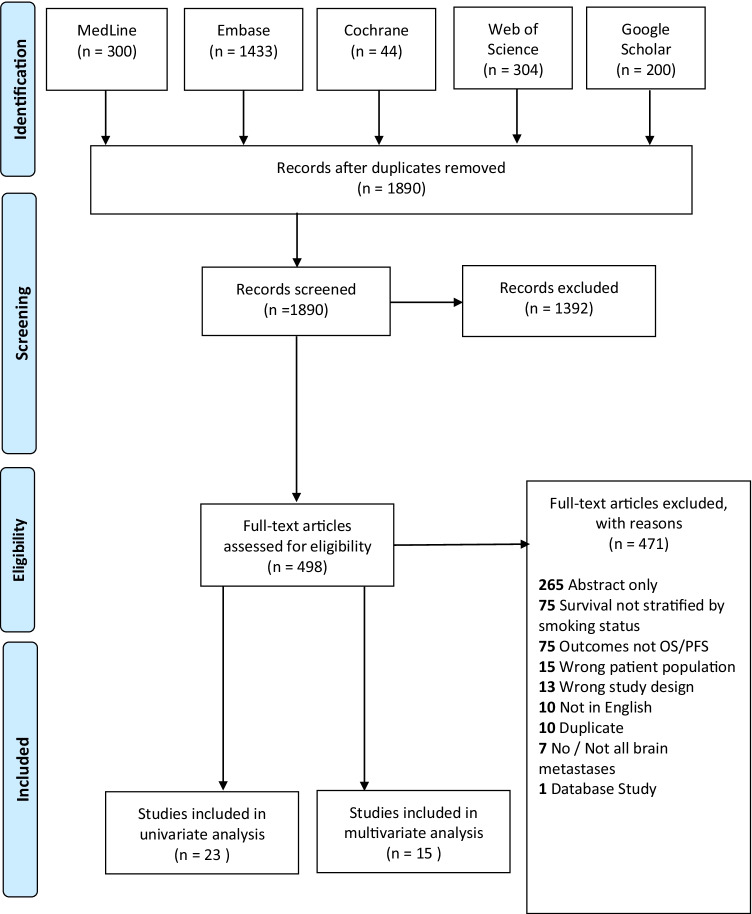
Prisma flowchart

The search strategy returned 1890 articles following removal of duplicated papers. After title and abstract followed by full-text screening, fifteen studies [5, 6, 8, 12, 13, 18, 21, 23–25, 31, 32, 41, 50, 52] included data on multivariate HR (as opposed to univariate), meeting our inclusion criteria, and were included in our review and meta-analysis (Fig. 1). Papers reporting data on univariate HR [2, 8, 9, 12, 13, 16, 22–25, 29–33, 41, 42, 44, 48–52] (*n* = 23) were pooled in a sensitivity analysis and results can be found in supplementary materials. Of the 15 studies included, 13 were retrospective cohort studies, and two were prospective cohort studies. The mean study duration was 110.67 months, and the mean follow-up duration for OS and PFS was 17.11 months (Table 1). The most common covariates adjusted for in the multivariate models were age (*n* = 9) and sex (*n* = 7); the minimum number of covariates in a model was 3 [21, 26] and the maximum was 11 [32]. Of the total 3094 pooled participants, 63.2% (*n* = 1956) were male. Smoking status was reported in 87.0% (*n* = 2692) of patients—for the proportion that was reported, 64.2% (*n* = 1727) were current or past smokers and 35.8% (*n* = 965) had no smoking history. The histology of 48.2% (*n* = 1492) of the primary lung carcinomas was adenocarcinoma (Table 2).

**Table 1:** Characteristics of included studies

Study number	Author (year)	Country	Study design	Sample size/# of deaths (if provided)	Study duration (months)^1^	Follow-up duration (months)^2^
1	Du T, 2020	China	Retrospective cohort	144	125	10
6	Zhuang Q, 2020	China	Retrospective cohort	250/230	120	18.9
2	Li YD, 2019	US	Retrospective cohort	125	41	-
3	Chen CH, 2019	Taiwan	Retrospective cohort	141	60	20.3
4	Hendriks LEL, 2019	The Netherlands France	Prospective cohort	255	66	15.8
5	Lu F, 2019	China	Retrospective cohort	206/97	84	22.7
7	Inal A, 2018	Turkey	Retrospective cohort	698	159	-
8	Kim IA, 2018	South Korea	Retrospective cohort	142	131	-
9	Kobayashi H, 2018	Japan	Retrospective cohort	59/39	360	17.9
10	Byeon S, 2016	Korea	Retrospective cohort	121	107	18.4
11	Zhang Q, 2016	China	Retrospective cohort	43	28	-
12	Duell T, 2015	Germany	Prospective cohort	118/103	96	8.6
13	Cai L, 2014^a^	China	Retrospective cohort	178	132	28
14	Sekine A, 2014	Japan	Retrospective cohort	197	91	10.5
15	Kim J, 2013	South Korea	Retrospective cohort	313	60	-

^1^Study duration refers to time period in which patients were included in the study. ^2^Follow-up refers to the median follow-up time for patients

**Table 2:** Patient characteristics

Author (year)	Gender (%male)	Age (mean years)	Smoking status	Number of brain metastasis	Histology	Mutation	Covariates in multivariate analysis
Du T, 2020	62.5	59	Yes: 74 No: 70	1: 36 (25%) ≥ 2: 108 (75%)	Adenocarcinoma: 76 SCLC: 44 Squamous: 24	-	Sex, age, smoking status, lung cancer histology, extracranial metastasis status, RPA class, DS-GPA class and BMFI
Zhuang Q, 2020	93.6	57.5	Yes: 166 No: 84	-	SCLC: 250	-	Age, ECOG score, smoking status, biologically effective dose (BED) in brain, pleural effusion
Li YD, 2019	45.6	64.8	Never: 23 < 30 PY: 40 ≥ 30 PY: 62	1: 33 (23.4%) 2–3: 15 (10.6%) > 3: 93 (66%)	Adenocarcinoma: 96 Squamous: 13 Large-cell: 7 Small-cell: 8	EGFR: 19 KRAS: 31 ALK: 3	Age, sex, smoking status, ECOG performance score, histology, treatment-naïve at SRS, immunotherapy with SRS, resection, absolute lymphocyte count, neutrophil–lymphocyte ratio
Chen CH, 2019	37.6	64.5	Current: 17 Ex-smoker: 17 Non-smoker: 107	-	NSCLC: 141	EGFR: 141	WBRT, sex, lung surgery, brain surgery, smoking status
Hendriks LEL, 2019^a^	62.0	58.5	Current: 103 Former: 122 Never: 16 Unknown: 14	1: 53 (25.7%) 2–3: 31 (15%) > 3: 122 (59.2%)	Adenocarcinoma: 199 Squamous carcinoma: 38 NSCLC, other: 18	EGFR: 10 ALK rearrangement: 1 KRAS: 67 BRAF: 4	Age, smoking status, histology, number of organs with metastasis, immune checkpoint inhibitor line, WHO performance status, use of corticosteroids, brain metastases
Lu F, 2019^b^	57.8	≤ 49: 78 ≥ 50: 126	Never smoker Current/ex-smoker	-	Adenocarcinoma: 183 Non-adenocarcinoma: 23	EGFR: 62	Age, smoking status, CCRT, CT after BRT, EGFR-TKI therapy, NSCLC-BMs months, neurologic symptoms, RPA class, GPA scores, WBRT scheme, BRT method
Inal A, 2018	86.4	< 65: 560 > 65: 138	Ever: 510 Never: 122 Unknown: 66	-	Adenocarcinoma: 396 Squamous cell carcinoma: 116 Other: 14 NSCLC NOS: 172	-	Smoking status, extracranial metastases, neurosurgical resection
Kim IA, 2018	35.2	65	Heavy: 17 Moderate: 22 Light: 12 Never: 91	-	Adenocarcinoma: 142	EGFR: 142	Age, sex, performance status, stage, timing of EFDFR-TKIs, cumulative smoking dose
Kobayashi H, 2018	76.3	61.3	Ever: 40 Never: 19	-	Solid: 34 Papillary: 13 Acinar: 11	EGFR: 14	Location, smoking status, EGFR mutation
Byeon S, 2016	31.4	58.2	Current: 6 Ex-smoker: 22 Never: 93	-	NSCLC: 121	EGFR: 121	Age, sex, smoking status, EGFR mutation, EGFR TKI, BM lesions, extracranial metastasis, ECOG performance status, co-existing leptomeningeal carcinomatosis
Zhang Q, 2016	44.2	55.75	Current: 11 Never smoker: 32	-	Adenocarcinoma: 42 Adenosquamous carcinoma: 1	EGFR: 43	EGFR mutation, smoking history, line of TKIs and ECOG performance status
Duell T, 2015^a^	52.5	59.75 ± 9.97	Ever: 83 Never: 35	-	Adenocarcinoma: 77 Squamous cell carcinoma: 23 Undifferentiated: 18	-	Age, sex, Karnofsky score, smoking status, metastases adrenal gland, metastases adrenal gland, metastases liver, systemic therapy, radiotherapy, neurological symptoms at diagnosis
Cai L, 2014^c^	63.8	59.5 ± 8.43	Ever: 82 Never: 96	< 3: 159 (56.4%) > 3: 123 (43.6%)	NSCLC: 282	EGFR: 55	Extracranial lesions, smoking status, BM number, BM size, T staging, N staging
Sekine A, 2014	57.9	-	Ever: 124 Never: 73	-	Adenocarinoma: 82	EGFR: 89	Sex, smoking status, ECOG PS, neurological symptoms, cranial radiotherapy, previous chemotherapy, number of brain metastases
Kim J, 2013	42.8	59.95 ± 10.80	Current: 39 Ex-smoker: 115 Never: 133 Unavailable: 26	-	Adenocarcinoma: 199 Squamous cell carcinoma: 36 Others: 71	-	Number of metastases, histologic type, age at diagnosis of brain metastasis, smoking status, treatment on primary lung cancer lesion, whether brain was only site of metastasis or not, treatment of brain metastasis

*RPA* recursive partitioning analysis; *DS-GPA* diagnosis-specific graded prognostic assessment, *BMFI* brain metastasis–free interval, *ECOG* Eastern Cooperative Oncology Group, *SRS* stereotactic radiotherapy, *WBRT* whole brain radiotherapy, *CCRT* concurrent chemoradiation, *BRT* brain radiotherapy, *EGFR TKI* epidermal growth factor receptor tyrosine kinase inhibitors, *GPA* graded prognostic assessment, *BM* brain metastasis. ^a^Although Hendriks et al. (2019) and Duell et al. (2015) provided information on the number of ever and never smokers, the multivariate HR was calculated as yes vs. no smokers. ^b^Lu et al. (2019) did not provide the number of patients in each smoking category. ^c^Sample size for Cai et al. (2014) only includes the non-tyrosine kinase inhibitor (TKI) group as the TKI group had no HR provided for smokers vs. non-smokers

### Overall survival

**Fig. 2:**
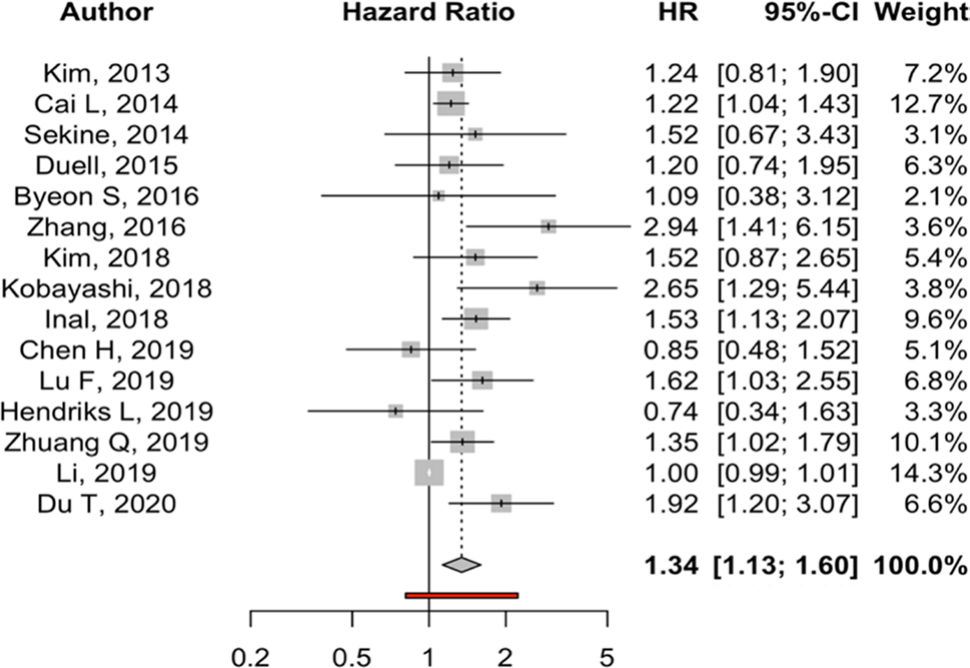
Forest plots showing pooled multivariate HR and 95% *CI* for all studies that compared overall survival comparing smokers vs. non-smokers lung carcinoma BM patients. Each study is shown by the point estimate of the hazard ratio and 95% confidence intervals (extending lines). The diamond centre represents the estimated pooled hazard ratio and width represents its 95% confidence interval (labelled total)

Fifteen studies, with a total of 2915 patients with brain metastases, met our inclusion criteria. The pooled multivariate HR for overall survival was greater amongst smokers than non-smokers (HR: 1.34, 95% *CI* 1.13, 1.60; 15 studies, *I*^2^: 72.1%; p-heterogeneity: < 0.0001) (Fig. 2). This suggests that death rates in BM patients in the smoking group were 34% higher than those in BM patients in the non-smoking group. The HRs were found to have a high level of heterogeneity.

### Subgroup analysis

**Fig. 3:**
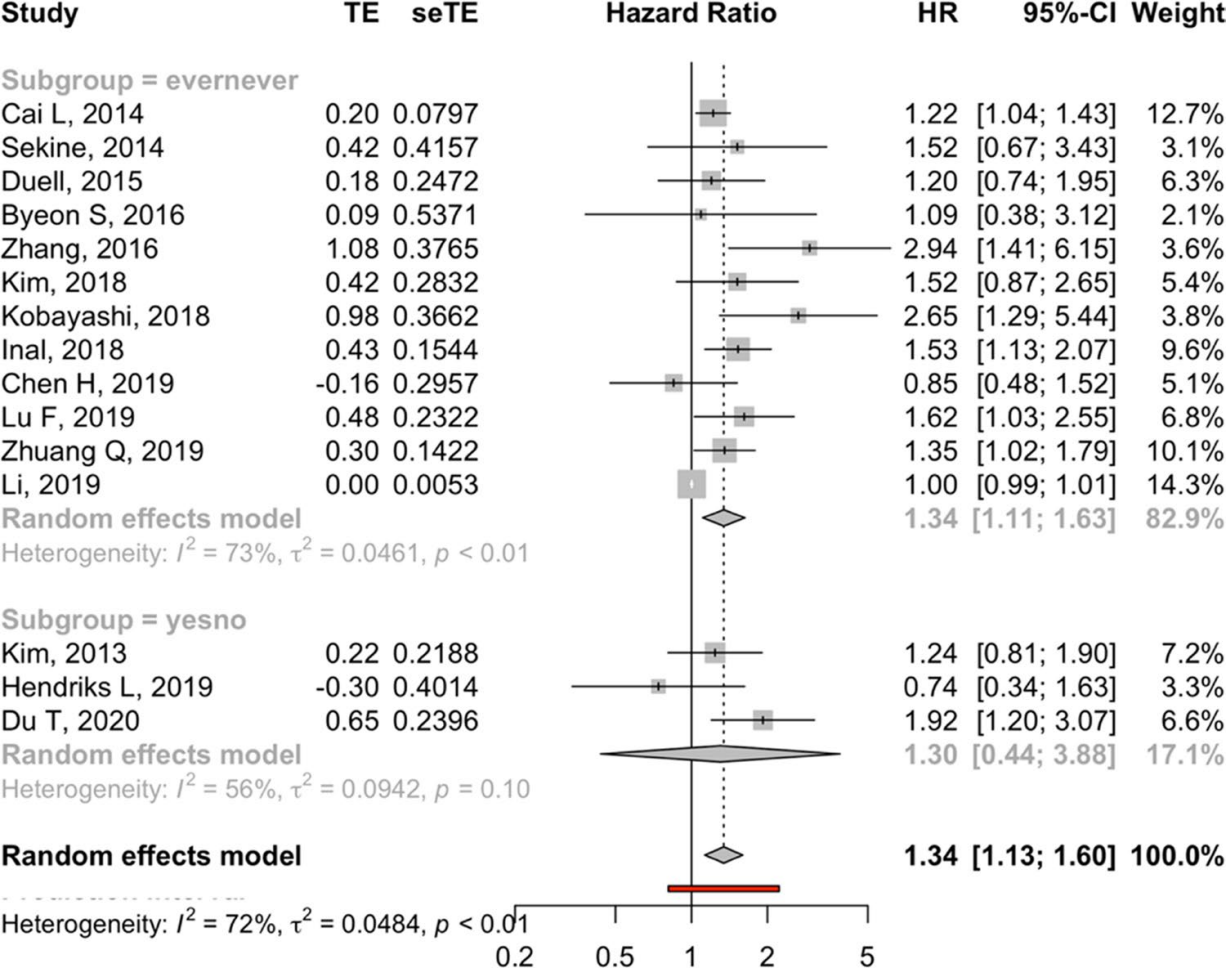
Forest plots showing HR and 95% *CI* across all studies comparing overall survival comparing smokers vs. non-smokers lung carcinoma BM patients, stratified by smoking status definition (ever vs. never; yes vs. no)

A subgroup analysis was performed to tease out heterogeneity in how smoking was reported across studies. Studies reporting smoking as ever/never had significant results showing smoking to be associated with an increased risk of death (HR: 1.34, 95% *CI*: 1.11, 1.63; 12 studies; *I*^2^: 73%; p-heterogeneity < 0.01). Studies reporting smoking as yes/no also displayed that smoking increased the risk of death, but these results were not statistically significant (HR: 1.30, 95% *CI*: 0.44, 3.88; 3 studies; *I*^2^: 56%; p-heterogeneity: 0.10) (Fig. 3).

### Progression-free survival

**Fig. 4:**
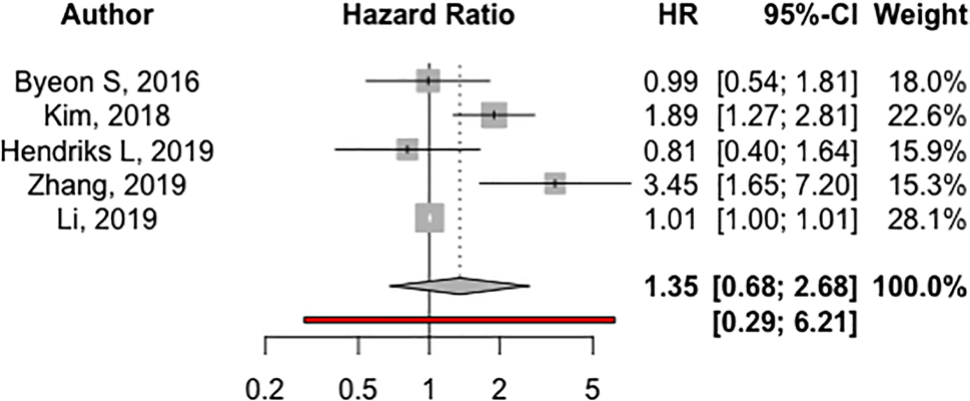
Forest plots showing HR and 95% *CI* across all studies that compared progression-free survival comparing smokers vs. non-smokers lung carcinoma BM patients

The pooled multivariate HR for PFS demonstrated worse outcomes amongst smokers (HR: 1.35, 95% *CI* 0.68, 2.68; 5 studies; *I*^2^: 80.8%; p-heterogeneity: 0.0003), when compared to non-smokers; however, this analysis did not reach statistical significance (supplementary materials Fig. 4) (Fig. 4). Stratifying by how smoking was reported was not feasible due to the paucity of studies in each category.

### Sensitivity analysis

Rerunning our multivariate analysis excluding the outlier result (Li 2019) resulted in a pooled multivariate HR of 1.39 (95% *CI* 1.18, 1.64; 14 studies, *I*^2^ = 28.6%; p-heterogeneity: 0.15). This was similar to our results including the outlying HR (supplementary materials Fig. 1). Our sensitivity analysis pooling univariate studies yielded a pooled univariate HR of 1.14 (95% *CI* 1.05, 1.24; 23 studies, *I*^2^: 42%; p-heterogeneity: 0.02), which was consistent with our multivariate results (supplementary materials Fig. 2). Subgroup analysis was similarly consistent with the ever/never subgroup showing smoking to be associated with a significant increased risk of death (HR: 1.17, 95% *CI* 1.06, 1.30; 18 studies; *I*^2^: 53.1%; p-heterogeneity: < 0.01). Studies reporting smoking as yes/no showed that smoking was associated with a non-significant increase in death (HR: 1.05, 95% *CI* 0.88, 1.27; 7 studies; *I*^2^: 0%; p-heterogeneity: 0.55) (supplementary materials Fig. 3). Similarly, sensitivity analysis of PFS also suggested that smoking was associated with an increased risk of mortality (HR: 1.33, 95% *CI* 0.99, 1.79, *I*^2^: 70.1%; p-heterogeneity: 0.0027), but this was not statistically significant. Further subgroup analysis was not possible due to paucity of studies in each category.

### Bias evaluation

**Fig. 5:**
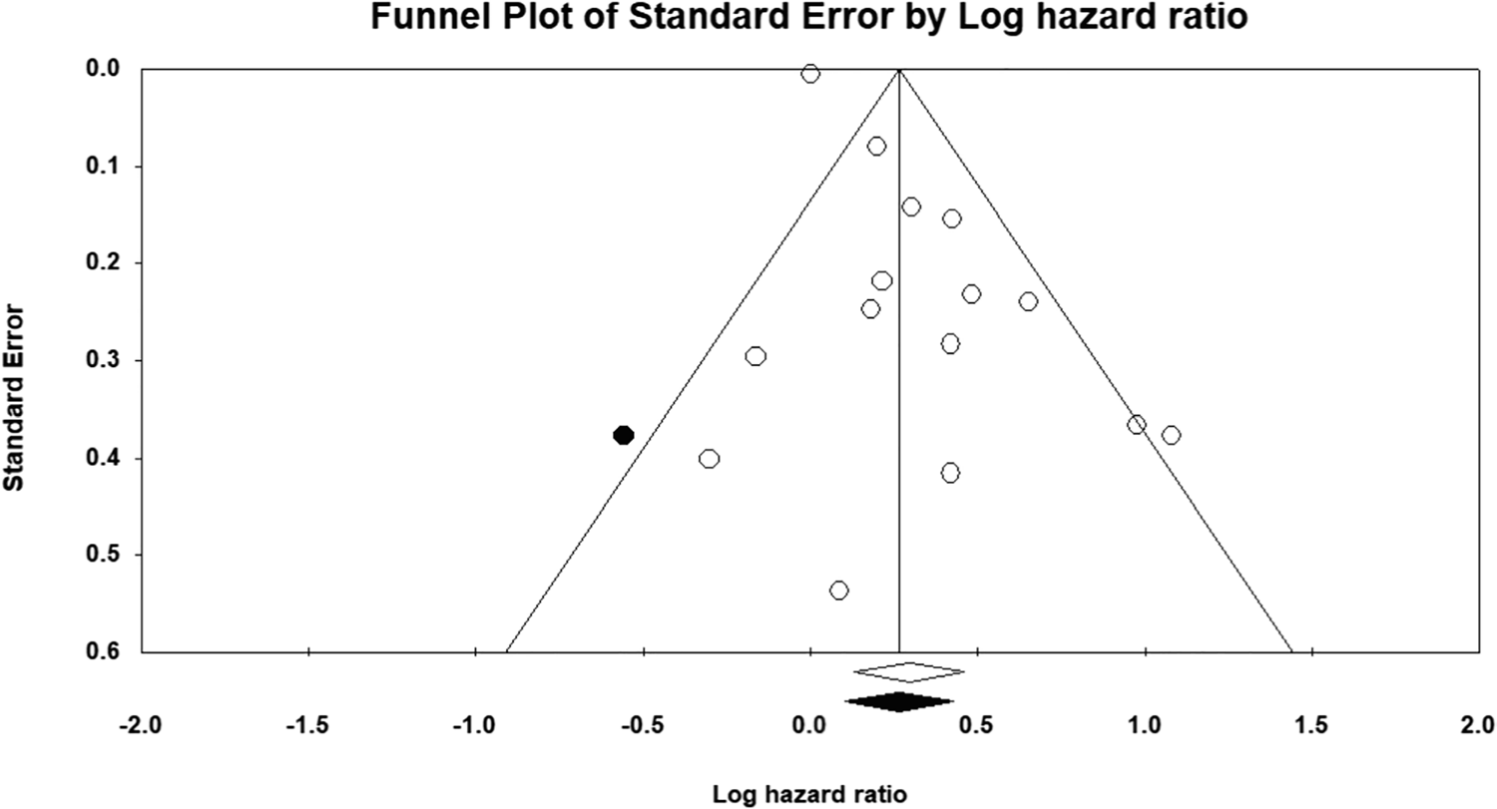
Funnel plot of standard error by log hazard ratio

Small study bias was present on visual examination of the funnel plots. Eggers’ test confirmed the presence of funnel plot asymmetry (*p* = 0.001) (Fig. 5). The trim-and-fill method was attempted using the random effects model. One study was trimmed. The adjusted hazard ratio significantly indicated that smoking was associated with an increased risk of death (HR: 1.30, 95% *CI* 1.11, 1.53). The quality score for the observational studies ranged from 6 to 8 out of a total of 9 points on the Newcastle–Ottawa Scale, with a median score of 7, indicating that the quality of the studies was borderline fair/good (Supplementary materials Table 1).

## Discussion

This meta-analysis confirmed that smoking was statistically significantly associated with an increased risk of death in patients with brain metastases from lung cancer. Moreover, our subgroup analysis revealed that this result was consistent whether the smoking status was categorised as ever/never or yes/no. However, the yes/no subgroup yielded statistically insignificant results, which could be due to the small sample size (3 studies) or the fact that the “no” category could include patients who recently stopped smoking, leading to an underestimation of the associated risk.

Our findings are clinically significant for the management of patients with brain metastases. Previous studies and clinical empiricism have suggested that there is a lack of emphasis on smoking cessation for patients with brain metastases given their short life-expectancy [42].

This meta-analysis quantitatively assessed that BM patients that were non-smokers have longer median OS. Results for PFS did not reach significance, likely due to the smaller sample size. Several previous studies that examined the effect of smoking on cancer patients have restricted the examined cohort to non-metastatic patients [36, 37]. However, there is previous literature examining the effect of smoking on different types of metastases which have found similar results to ours [17, 27].

Our sensitivity analysis of univariate HR was consistent with our multivariate results. Controlling for other factors in the multivariate analysis resulted in the pooled effect size being more strongly suggesting the risk of death was greater amongst smokers. The overall quality assessment of the studies was noted to be “good”. However, it is important to note that three studies—Hendriks et al. (2019) [18], Inal et al. (2018) [21] and Kim et al. (2013) [24]—reported smoking status to be unknown for 14/255, 66/698 and 26/313 patients, respectively. Another key aspect limiting the quality of these studies is that smoking was self-reported by the patients and the fact that some studies did not explicitly categorise patients as current, former and never smokers, and instead lumped former and never smokers together.

Several biologic processes explain the link between smoking and poor outcomes in metastatic patients. Recently, a 2019 study found that smoking increased the incidence of brain metastases in lung cancer patients and that nicotine had a critical impact on promoting metastatic development by skewing microglia to alternatively activated phenotype and suppressing their role in innate immunity [47]. This ultimately enhances metastatic tumour growth. It is well-established that smoking alters biologic pathways of cancer resulting in greater proliferation, migration, invasion, angiogenesis and activation of pro-survival cellular pathways [42, 43]. This leads to a more malignant tumour phenotype and can therefore worsen outcomes of patients. This further supports our findings that smoking increases the risk of death in patients with brain metastases and stresses the importance of advocating smoking cessation for these patients.

Only three out of the 15 included studies reported on EGFR mutation status. Previous papers have found an association between smoking and a lower number of EGFR mutations [38, 45]. However, Tseng et al. [45] found that smokers had a shorter median overall survival (OS) amongst both EGFR-mutant and EGFR-wild type patients (17.8 vs. 21.1 months, and 7.9 vs. 11.4 months, respectively; both *p* < 0.001). If there is indeed a relation between smoking and EGFR mutation status, this suggests that we should not adjust for EGFR mutation as it is a downstream consequence of the exposure (smoking) and we would be over adjusting. Future studies should report on mutational status in order to further understand the relation of smoking to overall survival with respect to mutational status.

Additional questions must be addressed in order to achieve a more in-depth understanding of the impact of smoking on patients with lung cancer and metastases. Interestingly, a recent meta-analysis comparing lung cancer patients who quit smoking at or around diagnosis or during treatment to those who continued smoking concluded that quitting smoking was significantly associated with improved overall survival [7]. Future studies should report whether patients have quit smoking after diagnosis/around treatment in order to enable a meta-analysis that can analyse whether quitting smoking is indeed beneficial for patients with brain metastases. Notably, only four out of 15 studies [6] explicitly defined that overall survival counted cancer-related deaths only; future studies conduct a competing risk analysis where they report cause-specific hazards or sub-distribution hazards for cancer- or non-cancer-related causes of deaths [28]. Studies should also report the number of deaths from each cause.

### Strengths and limitations

Limitations of this study include limiting our search to studies published in English. Additionally, there was heterogeneity in the way smoking status was reported (ever vs. never, yes vs. no) and we were unable to analyse the difference between current smokers and former smokers; nevertheless, we stratified our results by the way smoking categories were reported. Moreover, only two studies, Zhang et al. 2016 [50] and Kim et al. 2013 [24], provided data on current, past and never smoking. Heterogeneity in the definition of smoking status is an important limitation for future studies to address because the “no” category could include participants who were both never or former smokers, which can confound the estimated association. There was also a lack of consistent reporting of point estimates with only a portion of studies reporting multivariate HR along with univariate HR.

However, this study also had several strengths. We were able to demonstrate that smoking cessation improved survival outcomes in patients with lung carcinoma brain metastases. A strict protocol was adhered to in performing the meta-analysis. We were meticulous in extracting MV-adjusted HRs in our main analysis and considered univariate HRs in a sensitivity analysis, as these would be biassed. Additionally, we teased out the heterogeneity in which smoking was reported. Furthermore, we analysed 15 studies with a sample size of 3094, which allowed good power in detecting a statistically significant hazard ratio, which favoured non-smokers.

## Conclusion

In conclusion, our meta-analysis suggests that a smoking history has detrimental effects even in a progressive phase of malignancy. There were similar results for PFS but this did not reach statistical significance. Future studies should use a standardised way of reporting smoking status, such as never, past and current smokers, to facilitate analysis on how smoking cessation after diagnosis impacts survival. Future studies should document whether patients have quit smoking after diagnosis/around treatment in order to determine if quitting smoking is indeed beneficial for patients with brain metastases. Additionally, a time-to-event analysis would be beneficial to compare brain metastases between never, past and current smokers.

Supplementary information.

## Supplementary Information

Below is the link to the electronic supplementary material.

Supplementary file1 (DOCX 2288 KB)

## Data Availability

The review data generated during and/or analysed during the current study are available from the corresponding author on reasonable request.

## Code availability

The review data generated during and/or analysed during the current study are available from the corresponding author on reasonable request.
