# Supplementary material for: The effect of smoking on survival in lung carcinoma patients with brain metastasis: a systematic review and meta-analysis
Source: Neurosurg Rev. 2022 Jul 14;45(5):3055–66. doi: 10.1007/s10143-022-01832-1 (PMC9492581; doi:10.1007/s10143-022-01832-1)
Supplement: Supplementary file 1 — Supplementary file1 (DOCX 2288 KB) [file 10143_2022_1832_MOESM1_ESM.docx]

**Supplementary Materials**

**Appendix 1. Search Strategy**

**(Performed 12/2020)**

**Pubmed**

((((Brain Neoplasms/ OR Brain/) AND (exp Neoplasm Metastasis/)) OR (((brain* OR cerebr* OR Pituitar* OR cranial* OR intracranial* OR skull) ADJ6 metasta*)).ab,ti.) AND ("Smoking"/ OR "Pipe Smoking"/ OR exp "Tobacco Smoking"/ OR "Nicotine"/ OR ((nicotine* OR smoking OR smoker* OR cigar*OR tobacco*).ab,ti.)))

**Embase**

('brain metastasis'/exp OR (('brain tumor'/exp OR brain/exp) AND ('metastasis'/exp OR 'central nervous system metastasis'/de OR 'distant metastasis'/de)) OR (((brain* OR cerebr* OR Pituitar* OR cranial* OR intracranial* OR skull) NEAR/6 metasta*)):ab,ti) AND ('smoking and smoking related phenomena'/exp OR 'smoking'/exp OR 'nicotine'/exp OR (nicotine* OR smoking OR smoker* OR cigar*OR tobacco*):ab,ti)

**Web-of-Science**

247 TS=(((brain* OR cerebr* OR Pituitar* OR cranial* OR intracranial* OR skull) NEAR/5 metasta*) AND (nicotine* OR smoking OR smoker* OR cigar*OR tobacco*))

**Cochrane**

25 ((((brain* OR cerebr* OR Pituitar* OR cranial* OR intracranial* OR skull) NEAR/6 metasta*):ab,ti) AND ((nicotine* OR smoking OR smoker* OR cigar*OR tobacco*):ab,ti))

**Google Scholar**

200 Brain metastases|metastasis|metastatic smoking|smoker|smokers|tobacco|cigars|cigarettes survival|risk|histology

**
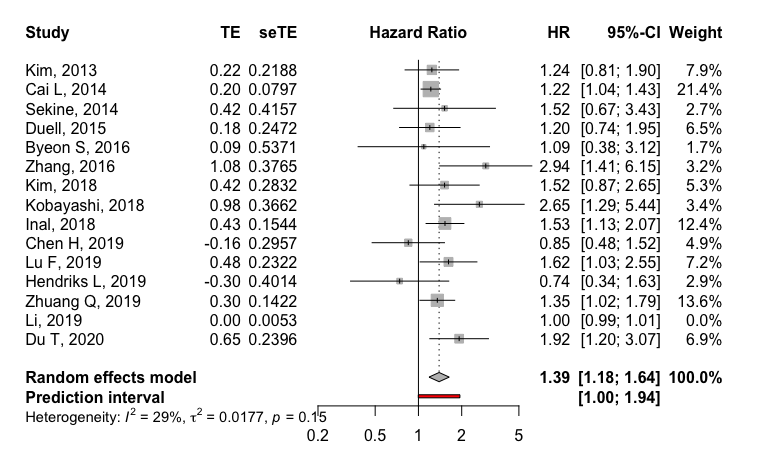
**

**Supplementary Figure 1. Forest plots showing pooled multivariate HR and 95% CI for multivariate overall survival comparing smokers vs. non-smokers lung carcinoma BM patients with the outlier (Li, 2019) omitted**


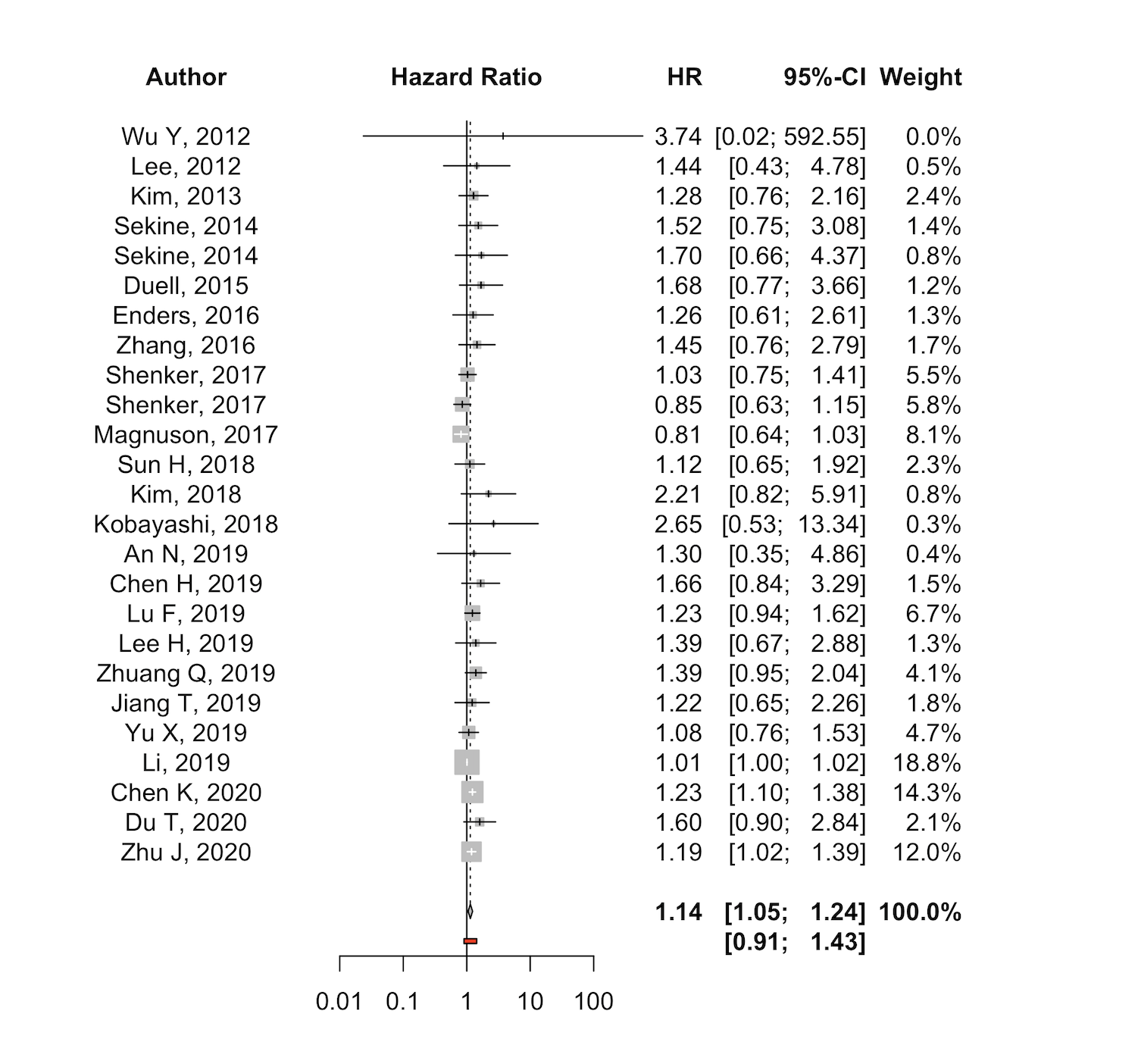


**Supplementary Figure 2. Forest plots showing pooled univariate HR and 95% CI for all studies that compared univariate overall survival comparing smokers vs. non-smokers lung carcinoma BM patients**


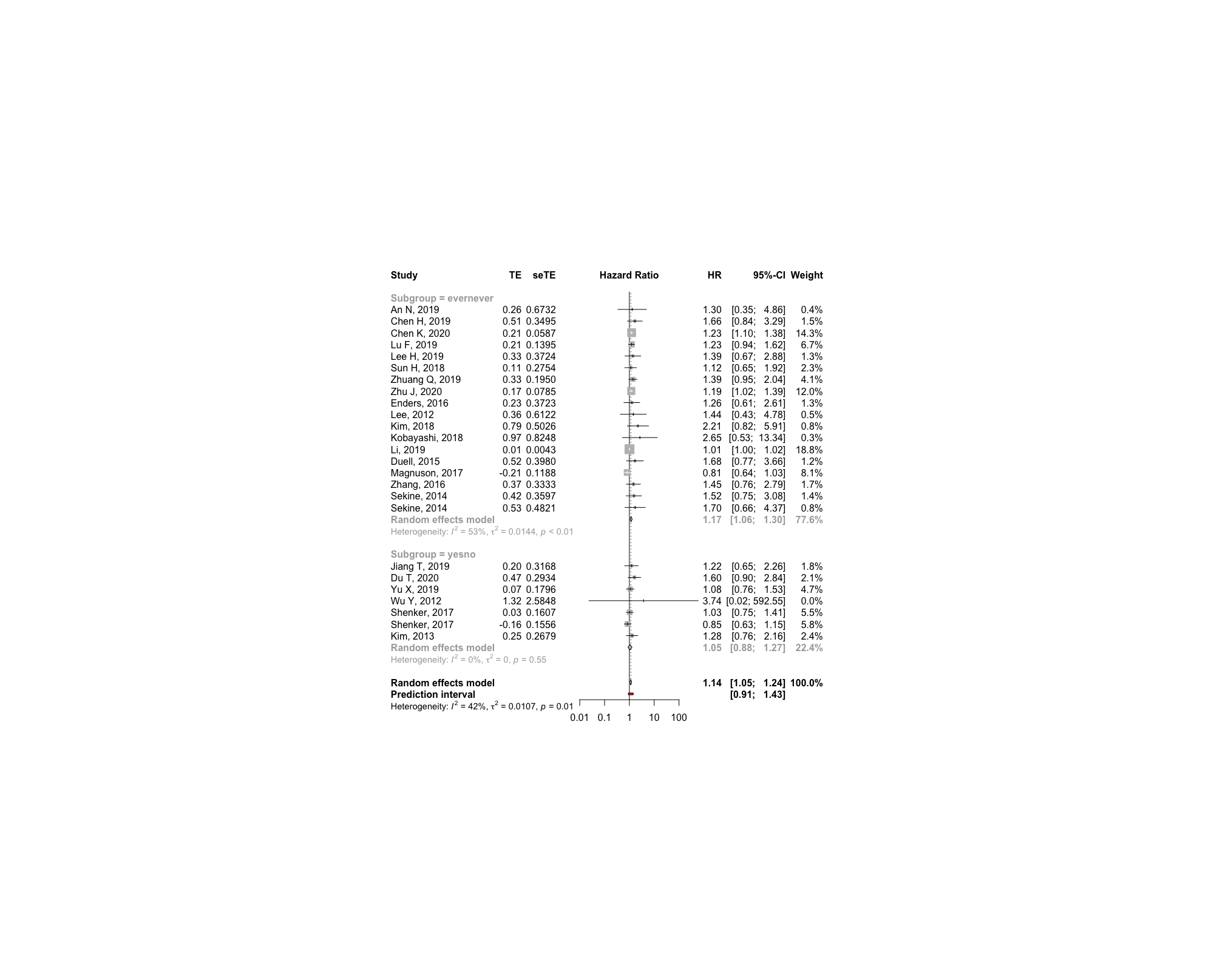


**Supplementary Figure 3. Forest plots showing pooled univariate HR and 95% CI across all studies comparing univariate overall survival comparing smokers vs. non-smokers lung carcinoma BM patients, stratified by smoking status definition (ever vs. never; yes vs. no).**

*Please note although the analysis pooled 18 studies for the ever/never and 7 studies for the yes/no subgroups, the actual number of papers was 17 and 6, as two papers had more than one group (with non-overlapping patient populations) that was eligible for inclusion in our analysis.*

**
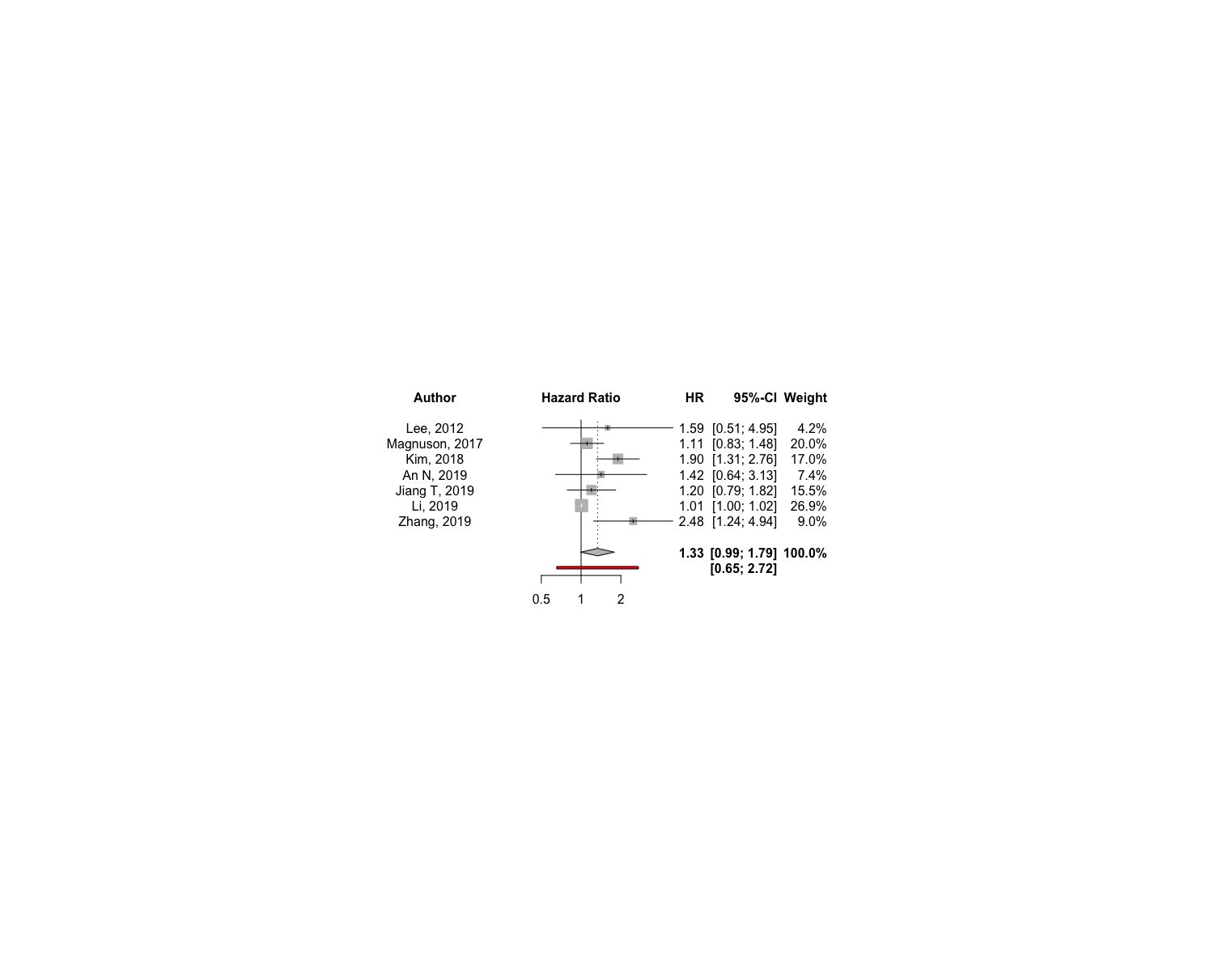
**

**Supplementary Figure 4. Forest plots showing pooled univariate HR and 95% CI across all studies comparing univariate progression survival comparing smokers vs. non-smokers lung carcinoma BM patients**

**Supplementary Table 1. Assessment Scores for Study Bias Using the Newcastle-Ottawa Scale**

|  | Selection | Comparability | Outcome | Total |
| --- | --- | --- | --- | --- |
| Prospective cohort studies | | | | |
| Hendriks LEL, 2019 | 2 | 1 | 3 | 6 |
| Duell T, 2015 | 3 | 1 | 3 | 7 |
| Retrospective cohort studies | | | | |
| Du T, 2020 | 2 | 1 | 3 | 6 |
| Zhuang Q, 2019 | 3 | 2 | 3 | 8 |
| Li YD, 2019 | 3 | 1 | 3 | 7 |
| Chen CH, 2019 | 2 | 1 | 3 | 6 |
| Lu F, 2019 | 3 | 1 | 3 | 7 |
| Inal A, 2018 | 3 | 1 | 3 | 7 |
| Kim IA, 2018 | 3 | 1 | 3 | 7 |
| Kobayashi H, 2018 | 3 | 2 | 3 | 8 |
| Byeon S, 2016 | 3 | 1 | 3 | 7 |
| Zhang Q, 2016 | 3 | 1 | 3 | 7 |
| Cai L, 2014 | 3 | 1 | 3 | 7 |
| Sekine A, 2014 | 3 | 1 | 3 | 7 |
| Kim J, 2013 | 3 | 1 | 3 | 7 |

***The New-Castle Ottawa Scale assesses the domains of subject selection (4 points), comparability ( 2 points) and assessment of outcome (3 points) for a total of 9 points. The score was interpreted as 0-3 points = “poor quality”, 4-6 points = “fair quality”, and 7-9 points = “good quality”.***
